# Dynamic Interplay between Microbiota Shifts and Differential Metabolites during Dairy Processing and Storage

**DOI:** 10.3390/molecules29122745

**Published:** 2024-06-09

**Authors:** Yinan Zhang, Peng Yu, Fei Tao

**Affiliations:** 1 Key Laboratory of Milk and Dairy Products Detection and Monitoring Technology for State Market Regulation, Shanghai Institute of Quality Inspection and Technical Research, Shanghai 200233, China; 2 State Key Laboratory of Dairy Biotechnology, Shanghai Engineering Research Center of Dairy Biotechnology, Dairy Research Institute, Bright Dairy & Food Co., Ltd., Shanghai 200436, China; yupeng@brightdairy.com; 3 State Key Laboratory of Microbial Metabolism, and School of Life Sciences & Biotechnology, Shanghai Jiao Tong University, Shanghai 200240, China

**Keywords:** microbiota diversity, microbial metagenomics and metabolomics, metabolic pathway enrichment, correlation analysis of differential metabolites, dairy product quality

## Abstract

Due to the intricate complexity of the original microbiota, residual heat-resistant enzymes, and chemical components, identifying the essential factors that affect dairy quality using traditional methods is challenging. In this study, raw milk, pasteurized milk, and ultra-heat-treated (UHT) milk samples were collectively analyzed using metagenomic next-generation sequencing (mNGS), high-throughput liquid chromatography-mass spectrometry (LC-MS), and gas chromatography–mass spectrometry (GC-MS). The results revealed that raw milk and its corresponding heated dairy products exhibited different trends in terms of microbiota shifts and metabolite changes during storage. Via the analysis of differences in microbiota and correlation analysis of the microorganisms present in differential metabolites in refrigerated pasteurized milk, the top three differential microorganisms with increased abundance, *Microbacterium* (*p <* 0.01), unclassified *Actinomycetia* class (*p <* 0.05), and *Micrococcus* (*p <* 0.01), were detected; these were highly correlated with certain metabolites in pasteurized milk (*r* > 0.8). This indicated that these genera were the main proliferating microorganisms and were the primary genera involved in the metabolism of pasteurized milk during refrigeration-based storage. Microorganisms with decreased abundance were classified into two categories based on correlation analysis with certain metabolites. It was speculated that the heat-resistant enzyme system of a group of microorganisms with high correlation (*r* > 0.8), such as *Pseudomonas* and *Acinetobacter*, was the main factor causing milk spoilage and that the group with lower correlation (*r* < 0.3) had a lower impact on the storage process of pasteurized dairy products. By comparing the metabolic pathway results based on metagenomic and metabolite annotation, it was proposed that protein degradation may be associated with microbial growth, whereas lipid degradation may be linked to raw milk’s initial heat-resistant enzymes. By leveraging the synergy of metagenomics and metabolomics, the interacting factors determining the quality evolution of dairy products were systematically investigated, providing a novel perspective for controlling dairy processing and storage effectively.

## 1. Introduction

The microbiota of raw milk exhibits considerable complexity, with its composition subject to the influence of numerous factors. These include the geographical location of the pasture, the breeding environment, the season and timing of milk harvesting, the collection techniques, the storage temperature, and the duration of storage [1]. The microbiota present in milk significantly influences the quality and shelf life of various dairy products, including pasteurized, high-temperature sterilized, and ultra-high-temperature sterilized milk. Additionally, it plays a pivotal role in determining the flavor and quality of processed dairy products, such as cheese, butter, yogurt, and milk powder. Refrigeration and high temperature are the primary methods used to prevent the growth of microorganisms in raw milk [2]. Nonetheless, psychrotrophic microorganisms, heat-resistant microorganisms, and their heat-resistant proteases and esterases are uncontrollable factors during the subsequent processing and shelf life [3]. The alterations in microbiota throughout the processes of raw milk collection, refrigeration, and heat treatment have consistently represented a pivotal aspect of quality control within the dairy industry [4]. The investigation of the underlying mechanisms is crucial for maintaining the freshness of milk, regulating the sterilization process in liquid milk production, optimizing the manufacture of cheese and milk powder, and ensuring the safety of dairy products.

Extensive early studies, which are predominantly reliant on cultivation methods, have illuminated various aspects of milk microbial communities, including their types, sources, metabolites, correlations with quality, and control strategies. A significant focus has been placed on thermophilic bacteria and their enzymes such as lipases, proteases, and phospholipases [5,6,7,8]. The advent of culture-independent techniques, omics technologies, and advanced data analysis has enabled a more systematic exploration of the complexity of, diversity of, and biological activities inherent to milk microbiota [9]. Based on the quantification of psychrotrophic bacteria associated with the spoilage of refrigerated raw milk, bacterial genera were identified through diversity analysis of the high-variability region of 16-23S rRNA [10]. The development of high-throughput sequencing technology for 16S rRNA high-variability regions has provided relatively comprehensive community taxonomic information, which has been widely applied in the field of dairy microbial applied research. This includes correlations between specific dairy processing techniques and microbial community structures [11], an overview of the formation of microbial diversity in the production process of dairy enterprises [12], changes in microbial communities during the production process [13,14,15], and characteristic microbial communities in specific dairy products [16].

Metagenomic next-generation sequencing (mNGS) technology randomly fragments and reassembles microbial genomes into longer contiguous sequences to investigate microbial ecosystems. This approach circumvents the diversity loss incurred by the PCR bias and variability in amplicon sequencing, enabling the annotation of a species to the species level or even strain level, and it procures dependable information regarding biological function. It represents an efficient method for interrogating complex microbial systems. In-depth mNGS permits the comprehensive microbiota profiling of distinctive samples, allowing us to discern microbiota disparities, and furnishes the data necessary for elucidating the correlations between microbial functions and safety within dairy products. For instance, employing mNGS to discern predominant bacterial species’ variances between traditional natural cheeses and industrially produced ones, as well as investigating divergence in microbial community during storage, offers a theoretical foundation for cheese merchandising and storage conditions [17]. Through integrating deep mNGS with public database retrieval and annotation analysis, the identification of an extensive array of microbial species in various unique dairy fermentation products is accomplished, along with the annotation and analysis of the functional genes related to microbial nutrition, pathogenesis, bacteriophages, etc., underscoring the diversity and heterogeneity of the microorganisms present in fermented dairy [18,19]. Additionally, mNGS, coupled with functional analysis, explores alterations in microbial communities and the associated functional shifts throughout the refrigerated storage of raw milk [20], and resistance gene profiling predicts the resistance distribution patterns of dominant bacteria within dairy microbial communities [21].

The comprehensive investigation of microbiota necessitates the integration of transcriptomic, proteomic, and metabolomic data to thoroughly examine the functional mechanisms of the microbial communities present in dairy products. Metabolomic analysis integrates high-throughput and high-precision analytical techniques, compound databases, and statistical methodologies to identify and assess metabolites pertinent to other system parameters. This approach has been extensively employed across various disciplines, including physiology, pathology, pharmacology, animal nutrition, zoology, and botany. In the realm of raw milk and dairy product quality and safety monitoring, the application of metabolomics primarily delves into the testing of raw milk’s processing performance, milk nutrition research, milk biomarker research, and fermented milk processing research [22]. Studies based on multiple metabolomic detection platform technologies have revealed that the heat processing of pasteurized milk exerts a minimal impact on product quality; however, there are notable discrepancies in metabolites before and after refrigeration [23]. These findings lay the theoretical and technical foundation for investigating the correlation between the indigenous microbiota and metabolites in dairy products. Nevertheless, there remains a paucity of comprehensive reports on the integrated examination of alterations in both indigenous microbiota and metabolites within dairy products for use evaluating the quality of these products.

In the current study, we pioneered a synergistic approach aimed at thoroughly examining the synchronous fluctuations in both the microbiome and metabolome within dairy products as they undergo heat treatment and subsequent refrigeration. To achieve this, we harnessed the power of the innovative mNGS technology, leveraging the NovaSeq platform to characterize the shifts in microbiota. In tandem, we employed sophisticated analytical techniques including HPLC-MS/MS and GC-MS/MS to accurately determine the alterations in metabolite profiles. Furthermore, by deploying an integrative algorithmic framework, we meticulously dissected the complex interplay between the dynamic microbiota and the resultant metabolic products. This intricate analysis allowed us to find the pivotal microbial species that substantially influence the qualitative integrity of dairy products throughout their processing and storage stages. Our integrated multiomics strategy is an inspiring endeavor within the dairy industry, blending metabolomic and microbiomic methodologies. We anticipate that this exploration will pave a new way for the enhancement of food processing and preservation techniques, thereby offering invaluable insights to be leveraged in future food science research.

## 2. Results

### 2.1. General DNA Sequencing Observations

This study investigated samples of the same batch of frozen raw milk, refrigerated raw milk, refrigerated pasteurized milk, and refrigerated storage UHT milk, with the groups named Raw_fro, Raw_ref, Pas_ref, and UHT_ref, respectively. A total of 12 samples were subjected to mNGS with ref (n = 3), and the sequencing and assembly information are shown in Table 1. The results indicated that, after optimizing the host removal process, each sample produced reads, an original sequence length, contigs that were obtained through splicing and assembly, and an ORF sequence number that was predicted by the genes. The relevant data ensured the completeness and validity of the results.

A non-redundant gene set was constructed, and we performed gene statistical analysis and functional annotation for subsequent taxa (Figure S1; Tables S1–S3). Based on the Non-Redundant Protein Sequence Database (NR), the taxa annotations were obtained as follows: domain: 5, kingdom: 9, phylum: 67, class: 146, order: 325, family: 682, genus: 1448, and species: 5469 (Tables S4 and S5).

### 2.2. Diversity, Composition, and Difference of the Microbiota in Raw, Pasteurized, and UHT Milk after Storage

#### 2.2.1. The Diversity of the Processed Milk Samples Microbiota

**Figure 1:**
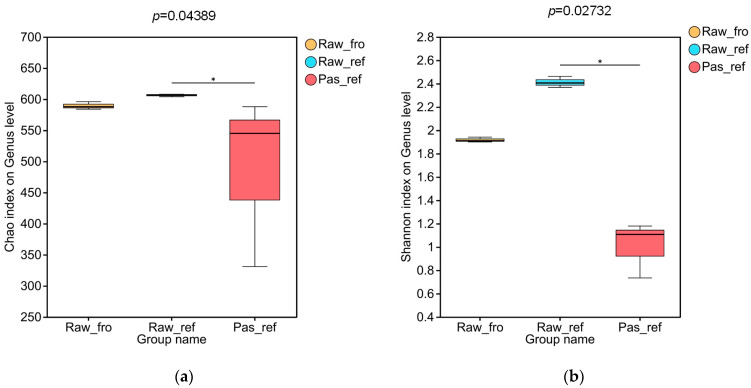
Alpha diversity indexes’ distribution for raw and pasteurized milk samples: (**a**) Chao1 richness index, and (**b**) Shannon diversity index. “*” represents a significant difference.

After calculating the operational taxonomic unit (OTU) estimates (Chao1 and Shannon index (Table S6)), significant differences were detected between the refrigerated raw milk group and the pasteurized milk group, compared with the UHT milk group (Figure 1). Studying the Chao1 index related to genus richness, we found that the diversity of the refrigerated raw milk sample community was the highest, followed by the refrigerated pasteurized milk, while that of the frozen raw milk was the lowest (Figure 1a). The Shannon index, which simultaneously evaluates genus richness and evenness, enhanced this difference in diversity (Figure 1b).

#### 2.2.2. The Composition of the Milk Microbiota

**Figure 2:**
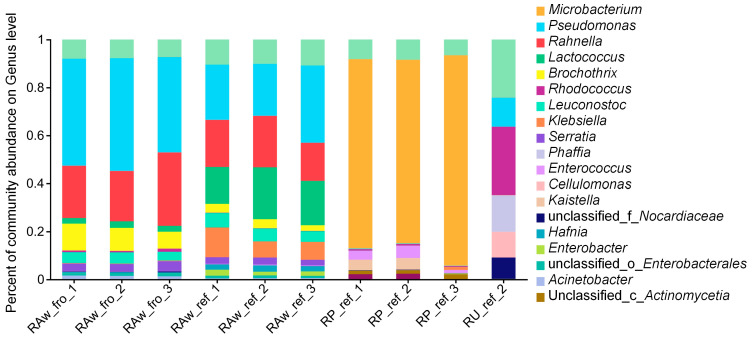
Community bar plot analysis of the microbiota composition at the genus level.

There were 16 genera and three unclassified taxa at the genus level with relative abundance greater than 0.01 in all milk samples (Figure 2). The dominant bacterial genera in the frozen raw milk samples (Raw_fro group) included *Pseudomonas* (46.54%), *Rahnella* (23.11%), *Brocothrix* (8.82%), *Leuconostoc* (4.01%), *Serratia* (3.17%), *Lactococcus* (2.44%), *Acinetobacter* (1.39%), *Rhodococcus* (1.06%) *Enterobacteriaceae* (1.03%), *Hafnia* (0.43%), *Klebsiella* (0.17%), and *Enterobacter* (0.06%), accounting for 92.25% of the total. The relative abundance of *Pseudomonas* (27.79%), *Lactobacillus* (18.32%), *Klebsiella* (8.86%), *Streptomyces* (5.01%), *Streptomyces* (3.16%), *Serratia* (2.53%), *Hafnibacteria* (2.20%), *Enterobacteriaceae* (1.96%), *Enterobacteriaceae* (0.98%), *Acinetobacter* (0.49%), and *Rhodococcus* (0.15%) remained the highest in the refrigerated raw milk samples (Raw_ref group), accounting for 89.52% of the total. The dominant genus in the pasteurized milk samples (Pas_ref group) was *Microbacterium*, accounting for 78.99% of the total. In addition, the relative abundance of *Enterococcus* (3.34%), *Kaitella* (2.83%), *Actinomycetia* (2.24%), *Chryseobacterium* (1.56%), *Micrococcus* (1.32%), *Erythrococcus* (0.68%), *Klebsiella* (0.44%), *Pseudomonas* (0.35%), *Enterobacteriaceae* (0.12%), *Enterobacteriaceae* (0.01%), and *Lactococcus* (0.01%) was relatively high. Meanwhile, the distribution of the microbial communities in the refrigerated UHT milk was uneven, and the DNA extracted from two of three samples did not meet the quality requirements for mNGS. One sample had the dominant bacterial genera of *Rhodococcus* (28.41%), others (24.28%), *Phaffia* (15.22%), *Pseudomonas* (12.16%), *Cellulomonas* (10.7%), unclassified_f_*Nocardiaceae* (8.92%), *Klebsiella* (0.09%), *Microbacterium* (0.07%), *Acinetobacter* (0.04%), and *Enterobacter* (0.01%).

#### 2.2.3. Microbiota Differences and Functional Differences of the Processed Milk Samples

**Figure 3:**
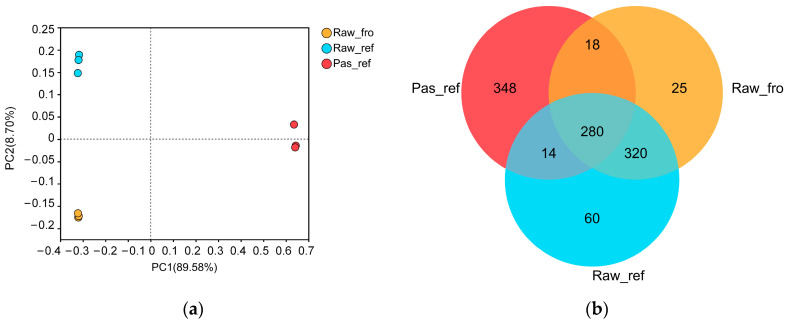
PCoA and Venn diagram of bacteria at the genus level: (**a**) PCoA analysis, (**b**) Venn analysis.

The results of principal co-ordinate analysis (PCoA) results for each sample, conducted at the genus level (Figure 3a), showed that the samples within the group were more clustered with slight differences, and the differentiation between the sample groups was more obvious (*p* = 0.001). There were significant changes in the genera of the raw milk, refrigerated raw milk, and refrigerated pasteurized milk. The Venn diagram (Figure 3b) showed that the number of genera in the refrigerated raw and pasteurized milk samples (674 and 660, respectively) was relatively increased compared with the number in the frozen raw milk (643). The genera in the refrigerated pasteurized milk were quite different from those in the refrigerated raw milk, since there were 348 specific genera in the group Pas_ref.

**Figure 4:**
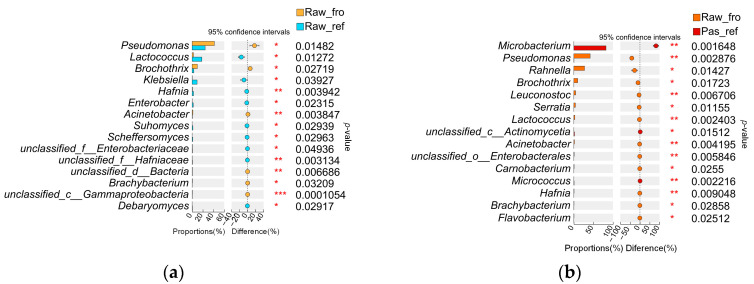
Welch *t*-test of bacteria at the genus level. (**a**)*t*-test of the genera in the Raw_fro group and Raw_ref group; (**b**) *t*-test of the genera in the Raw_fro group and Pas_ref group samples. “*” represents 0.01 < *p* ≤ 0.05, “**” represents 0.001 < *p* ≤ 0.01, “***” represents *p* ≤ 0.001.

The *t*-test analysis results (Figure 4a) showed that 240 of the genera were the genera that differed between the Raw_fro and Raw_ref groups (*p <* 0.05), among which *Acinetobacter*, *Hafnia,* and *Lentilactobacillus* showed significant differences (*p <* 0.01). Compared with the Raw_fro group, the relative abundance of *Pseudomonas*, *Brochothrix*, *Acinetobacter*, and *Bacillus* in the Raw_ref samples decreased. The relative abundance of *Lactococcus*, *Klebsiella*, *Enterobacterium*, *Hafina*, *Lentilactobacillus*, *Enterococcus*, *Candida*, *Lacticasebacillus*, and *Janthinobacillus* increased. The *t*-test analysis results showed that there were 382 differential bacterial genera between the Raw_fro and Pas_ref groups (*p <* 0.05), among which *Microbacterium*, *Pseudomonas*, *Leusonostoc, Lactococcus, Acinetobacter,* unclassified_c_*Enterobacterales, Micrococcus,* and *Hafnia* showed significant differences *(p <* 0.01) (Figure 4b). Compared with Raw_fro, the relative abundance of *Pseudomonas*, *Rahnella, Brochothrix, Leusonostoc, Serratia, Lactococcus*, *Acinetobacter, unclassified_o_Enterobacterales, Carnobacterium, Hafina*, *Brachybacterium,* and *Flavobacterium* in the Pas_ref group decreased. The relative abundance of *Microbacterium*, unclassified *actinomycetia* class, and *Micrococcus* increased. Via two-sample comparison, it was found that *Rhodococcus*, *Phaffia*, and *Cellulomonas* in the UHT milk sample increased compared to the frozen raw milk sample (Figure S2).

**Figure 5:**
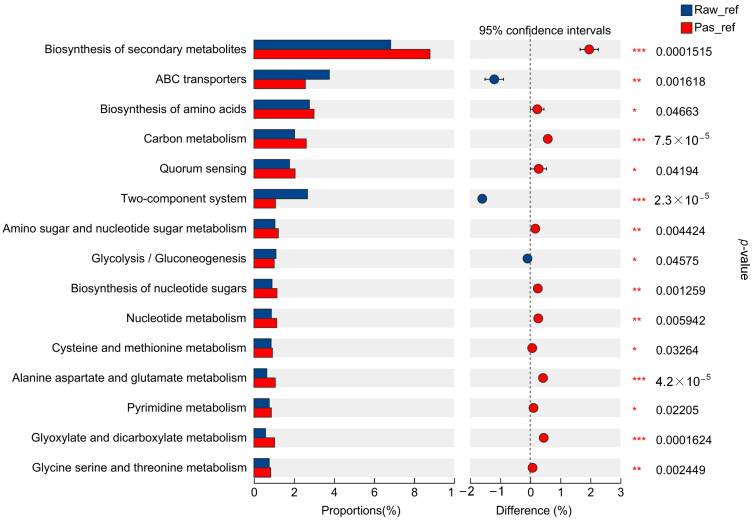
Welch *t*-test of differential KEGG pathways of Raw_ref group and Pas_ref group. * represents 0.01 < *p* ≤ 0.05, ** represents 0.001< *p* ≤ 0.01, *** represents *p* ≤ 0.001.

By comparing the metagenomic sequencing data of each sample with the Kyoto Encyclopedia of Genes and Genomes (KEGG) database (http://www.genome.jp/kegg/ (accessed on 30 March 2023)), the functional annotation information of the pathway was obtained (Table S7). The statistical analysis of the annotation results revealed a total of 46 pathways, the top 5 of which were related to carbohydrate metabolism, amino acid metabolism, membrane transport, metabolism of cofactor and vitamin, and energy metabolism (Figure S3). After the *t*-test analysis of the metabolism corresponding to the top 10 metabolites’ abundance percentage value between the Raw_ref group and Pas_ref group, the top three metabolic pathways corresponding to the positive effects were those relating to the biosynthesis of secondary metabolites; the degradation of valine, leucine, and isoleucine; and the biosynthesis of amino acids. The pathways corresponding to the negative effects were the two-component systems, the ABC transporters, and the phosphotransferase systems (PTS), respectively (Figure 5).

### 2.3. Identification and Comparison of Milk Metabolites from the Three Groups

The supernatants of the samples were taken from the same groups of Raw_fro, Raw_ref, Pas_ref, and UHT_ref. A total of 24 samples were subjected to chromatography-mass spectrometry analysis with ref (n = 6).

**Figure 6:**
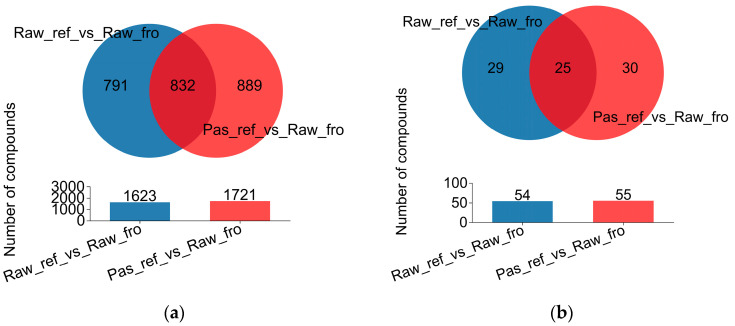
Metabolite Venn diagram map. (**a**) Metabolites analyzed using LC-MS; (**b**) metabolites analyzed using GC-MS.

LC-MS positive and negative-ionic-mode scanning analysis yielded 8572 and 5501 peaks that were quality-control (QC)-qualified (Figure S4), 1931 and 1716 identified metabolites, 1683 and 1666 metabolites in the library, and 637 and 577 metabolites in KEGG (Tables S8 and S9). After differential analysis between the refrigerated raw milk metabolite group and the frozen raw milk metabolite group, and between refrigerated pasteurized milk and the frozen raw milk, two differential metabolite sets of 1623 and 1721 compounds were obtained, respectively. A Venn diagram showed that there were 832 common metabolites and 791 and 889 specific metabolites in the two groups, respectively (Figure 6a). GC-MS in positive ion mode yielded 179 peaks that were QC-qualified (Figure S5), 179 identified metabolites, 60 metabolites in the library, and 58 metabolites in KEGG (Table S10). After differential analysis between the refrigerated raw milk metabolite group and the frozen raw milk metabolite group, and between the refrigerated pasteurized milk and the frozen raw milk, two differential metabolites sets of 54 and 55 compounds were obtained, respectively. The Venn diagram showed that there were 25 common metabolites and 29 and 30 specific metabolites in the two groups, respectively (Figure 6b).

**Figure 7:**
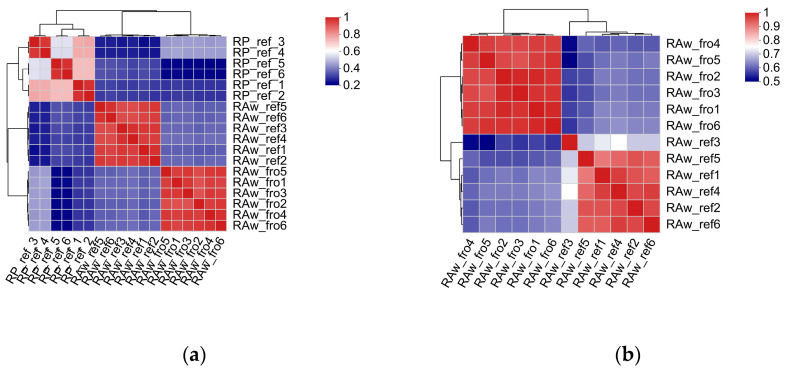
Metabolite correlation heat map. (**a**) Metabolites analyzed using LC-MS; (**b**) metabolites analyzed using GC-MS.

Quantitative analysis of the correlation between the variation degree of the metabolite composition and abundance among samples showed that the correlation among samples in the same group was relatively high. As illustrated in the correlation heat map (Figure 7), each group was located on different cluster branches; so, the sample repeatability met expectations. These results could be used for subsequent comparative analysis in groups.

**Figure 8:**
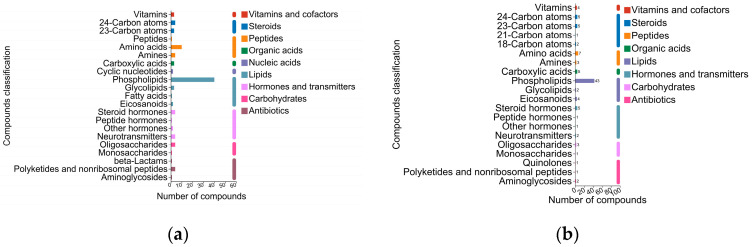
KEGG compound classification of differential metabolites. (**a**) Differential metabolites in Pas_ref_vs_Raw_fro group; (**b**) differential metabolites in Raw_ref_vs_Raw_fro group.

Comparing the identified metabolites to the KEGG compound database (https://www.kegg.jp/kegg/compound/ (accessed on 30 March 2023)), a summary of the metabolite classification and statistical plots was obtained (Figure 8). As shown in the statistical plots, the lipids, steroids, and peptides were the main differential metabolites, and they were closely related to milk nutrients such as protein and fat. Based on *t*-tests of metabolite abundance between the pairwise samples from different processing techniques (*p <* 0.05) and the Variable Importance in Projection (VIP) value of the Orthogonal Partial Least-Squares-Discriminant Analysis (OPLS-DA) model (VIP > 1), a total of 1128 differential metabolites between the refrigerated raw milk and the frozen raw milk (Raw_ref vs. Raw_fro group) were analyzed using LC-MS (Table S11), and 17 were analyzed using GC-MS (Table S12). For the metabolites analyzed using LC-MS, there were 462 differential metabolites that were likely related to protein and fat metabolism, according to the Human Metabolome Database (HMDB, https://hmdb.ca (accessed on 10 April 2023)) subclass. Similarly, from the LC-MS analysis, 519 out of 1624 differential metabolites were recognized between refrigerated pasteurized milk and frozen raw milk (comparison group: Raw_ref vs. Raw_fro) (Table S13). Additionally, 22 metabolites exhibiting significant differences were pinpointed from the GC-MS analysis (Table S14). The top five metabolites displaying the most significant variations were ranked in descending order based on the VIP scores derived from the OPLS-DA model, as presented in Table 2. The types and quantities of the differential metabolites in the refrigerated raw milk and the refrigerated pasteurized milk were quite different.

### 2.4. Metabolic Pathways Enrichment

**Figure 9:**
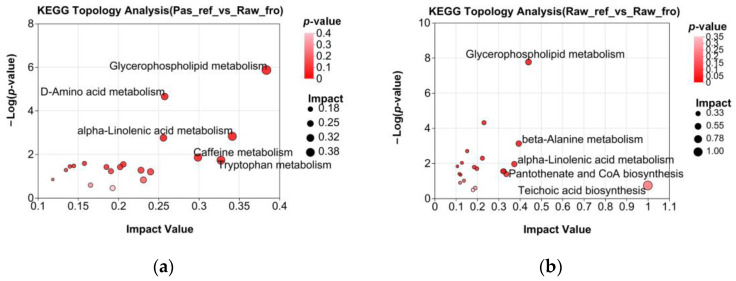
Enriched pathways in two pairwise groups are depicted as follows: (**a**) KEGG topology analysis of the differential metabolite sets between Pas_ref and Raw_fro; (**b**) KEGG topology analysis of the differential metabolite sets between Raw_ref and Raw_fro. In the figures, each bubble signifies a KEGG pathway. The horizontal axis indicates the relative importance of metabolites within the pathway and the magnitude of the impact value. The vertical axis represents the enrichment significance of the metabolites involved in the pathway, measured as −log10 (*p* value). The size of the bubble corresponds to the impact value, with larger bubbles indicating greater pathway importance.

KEGG pathway enrichment was performed on refrigerated raw milk and refrigerated pasteurized milk based on a comparison of the differential metabolite sets to the frozen raw milk. By analyzing the out-degree centrality of the metabolic pathways, a KEGG pathway topology map was obtained. From the graph (Figure 9), it can be concluded that for the refrigerated pasteurized milk, the top five important metabolic pathways were glycerophospholipid metabolism (map00564), alpha-Linolenic acid metabolism (map00592), tryptophan metabolism (map00380), caffeine metabolism (map00232), and D-amino acid metabolism (map00470). In contrast, for refrigerated raw milk, the top five metabolic pathways were teichoic acid biosynthesis (map00552), glycerophospholipid metabolism (map00564), beta-alanine metabolism (map00410), alpha-Linolenic acid metabolism (map00592), and pantothenate and CoA biosynthesis (map00770). The analysis of the important metabolic pathways reflected the changes in the microbial communities of the two samples during refrigeration. Compared with the refrigerated raw milk, the pasteurized milk microbiota exhibited relatively stronger lipid and amino acid metabolic activity during refrigeration, especially with the enhanced D-type amino acid metabolism unique to microorganisms and reduced cofactor metabolic activity.

### 2.5. Correlations between the Refrigerated Pasteurized Milk Microbiome and Differential Metabolites

**Figure 10:**
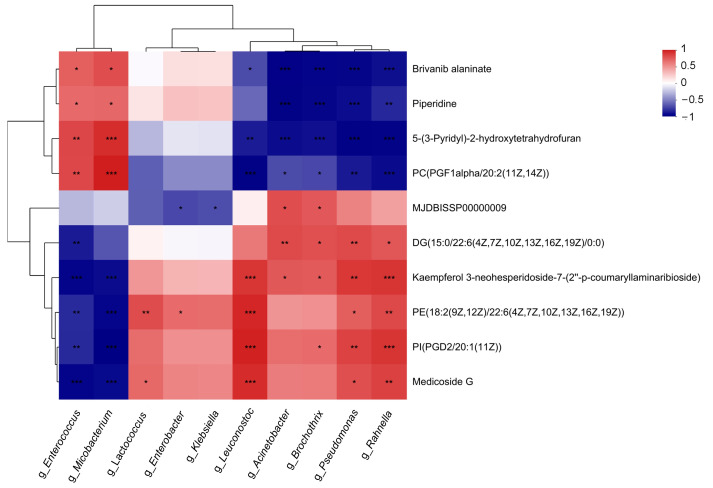
Correlation between differential metabolites (Top 10) and genus abundance (Top 10). * represented 0.01 < *p* ≤ 0.05, ** represented 0.001 < *p* ≤ 0.01, *** represented *p* ≤ 0.001.

The difference in the metabolite set of the Pas_ref_vs_Raw_fro group was intersected with that of the Raw_ref_vs_Raw_fro group to obtain a common differential metabolite set. The common differential metabolite set was subtracted from the differential metabolite set of the Pas_ref_vs_Raw_fro group to obtain the unique differential metabolite set of the refrigerated pasteurized milk (Table S15). Correlation analysis was conducted between the unique differential metabolite set and the microbial abundance data (Table S16) in all samples. We obtained a correlation heatmap, and cluster analysis was performed (Figure 10). Both the taxa and metabolites were divided into two distinct branches of differentiation. The second branch of taxa was obviously divided into two subbranches. It was found that the microorganisms in the first branch of taxa clustering were the genera with increased abundance, while the microorganisms in the second branch were the genera with decreased abundance. The correlation between microorganisms in the first subbranch of the second branch and high-abundance differential metabolites is relatively low. The microorganisms in the second subbranch are highly correlated with the metabolites. The compounds in the first branch of compound clustering were upregulated compounds present in refrigerated pasteurized milk, while the compounds in the second branch were downregulated compounds. The correlation between microbial genera (Top 50) and the metabolites (Top 50) was invariant (Figure S6, Table S17).

## 3. Discussion

All experimental samples were obtained by the freezing, refrigeration, or heating of the same batch of collected raw materials. This enabled a comprehensive investigation of the correlation between microbiota and their metabolites, as well as the comparison of community changes during processing. The richness and evenness of the microbiota in the refrigerated pasteurized milk were significantly reduced compared to the refrigerated raw milk samples (Figure 1). Due to the varying resistance of microorganisms to pasteurization, this result was consistent with our speculation. The corresponding differential metabolite analysis also revealed that there were multiple metabolites present in the refrigerated samples that were significantly different from those in the frozen samples, primarily phospholipids, sterols, amino acids, and peptides, which are closely related to the nutritional contents of dairy products (Table 2). Furthermore, the types of differential metabolites related to protein, amino acids, and lipid metabolism in the refrigerated pasteurized milk differed from those present in the refrigerated raw materials (Table 2). This suggests that microorganisms continue to grow and reproduce during the storage process after sterilization. Certain specific taxa are likely the primary factors causing nutritional changes during refrigeration. It was reported that Gram-positive spore-forming bacteria survive in pasteurized milk during refrigeration for 14–21 days [24]. A previous culture-independent analysis also indicated that the bacterial population of pasteurized milk is more diverse than previously appreciated; however, nonthermoduric bacteria present within these populations are likely to be in a damaged nonculturable form [25]. In this study, through the combined differential analysis of metagenomic data (Figure 4b), surviving microorganisms were more likely to be suggested for inclusion during refrigeration for longer than 30 days, including *Microbacterium*, a previously unclassified *Actinomycetia* class, and *Mircococcus*. Performing correlation analysis between microbial abundance and differential metabolite abundance in pasteurized milk, high-abundance microorganisms were found to be clearly clustered into two categories. Among them, *Microbacterium*, a previously unclassified *Actinomycetia* class, and *Mircococcus*, which displayed significantly increased relative abundance in pasteurized milk, were in the first category, while genera with decreased relative abundance were in the second category. This clustering analysis further demonstrates that genera with relatively increased abundance still maintain metabolic activity in pasteurized milk. The abundance of *Mriobacterium, Actinomycetia,* and *Micrococcus*, which increased significantly during refrigeration (Figure 4b) and differed from the reduced abundance of other genera in terms of metabolite correlation (Figure S6), likely contributed to the quality and extended shelf life of dairy products. Therefore, it is crucial to monitor the distribution of these microorganisms in raw milk. Previous studies based on cultivation have also pointed out that the proliferation of *Mriobacterium* and *Micrococcus* after separation from pasteurized milk is one of the reasons for the flavor defects and increased viscosity seen during a product’s shelf life [26,27]. Research has found that the main substances causing sensory changes in refrigerated pasteurized milk can be tentatively identified as a mixture of alcohols, aldehydes, ketones, and volatile acids. They are generated through various biochemical pathways as by-products of the metabolic activities (i.e., lipolysis and proteolysis) of microorganisms [28]. Through differential genera and metabolite correlation analysis, this study determined that the main metabolites positively correlated with proliferating microorganisms were amino acids, peptides, and analogues. In addition, the classes of pyrimidines and pyrimidine derivatives, estrane steroids, steroidal glycosides, carbonyl compounds, fatty acids and conjugates, pterins and derivatives, phenylpropanoids, polyketides, and glycerophosphoserines were also involved (Table 3). As anticipated, the microbial taxa and metabolites contained in the refrigerated raw milk and the refrigerated pasteurized milk were significantly different (Figure 4 and Figure 5), indicating that pasteurization can effectively control the proliferation of microorganisms, such as *Xanthobacterium*, *Pseudomonas*, *Lactococcus, Klebsiella*, and *Hafniella*. According to correlation analysis between microbial abundance and differential metabolite abundance in pasteurized milk (Figure 10 and Figure S6), the genera located in a cluster branch were different from microorganisms with increased relative abundance, which were clearly divided into two subbranches. On one of the subbranches, the genera, such as *Acinetobacter*, *Pseudomonas*, etc., were highly correlated with differential metabolites. It was reported that the thermophilic enzymes produced by psychrotrophic bacteria could preserve a significant portion of their activity (up to 75%), even after heat treatment. They are instrumental in the degradation process of proteins and fats in pasteurized milk, contributing to the formation of volatile organic compounds [29]. In contrast, genera such as *Lactococcus*, *Klebsiella*, and *Hafniella* showed minimal correlation with the differential metabolites. This strongly suggests that these microorganisms have a minor influence on the nutritional alterations in milk following pasteurization.

The indigenous microbiota was investigated using the primary identified taxa, including *Acinetobacter*, *Aerococcus*, *Brevibacterium*, *Corynebacterium*, *Enterococcus*, *Lactococcus*, *Staphylococcus*, *Streptococcus*, *Pseudomonas,* and *Weissella* [2,20,30,31]. *Pseudomonas*, *Acinetobacter*, *Lactococcus,* and *Xanthobacterium* are particularly concerning due to their elevated abundance in raw milk and strong ability to produce proteases and lipases. In this study, the main microbial genera identified in the raw milk samples were *Pseudomonas*, *Rahnella*, *Brocothrix*, *Leuconostoc*, *Serratia*, *Lactococcus*, *Acinetobacter*, *Rhodococcus Enterobacteriaceae, Hafnia*, *Klebsiella,* and *Enterobacter*. Notably, *Brocothrix*, a type of psychrotrophic bacteria commonly found in beef or chicken, was identified for the first time in raw milk. Research based on metagenomic sequencing results indicates that, with the extension of refrigeration time, there are significant changes in the quantity and population composition of microorganisms in raw milk. During the 72-h refrigeration period, the dominant bacterial community underwent a transition from the genera *Acinetobacter*, *Streptococcus*, *Acinetobacter*, and *Clostridium* to the genera *Xanthobacterium*, *Pseudomonas*, and *Lactococcus* [20]. In this study, compared with the frozen raw milk, the abundance of *Lactococcus*, *Klebsiella*, and *Hafniella* in the refrigerated raw milk increased (Figure 4a), indicating the evolution of bacterial genera over a refrigeration time longer than 30 days and presenting a different variety of psychrotrophic bacterial genera compared to 72-h refrigeration. Previous studies have shown that the rapid growth of psychrotrophic bacteria under refrigeration conditions does not have as much of an impact on the degree of spoilage in dairy products as expected [32]. However, as analyzed above, the heat-resistant enzyme systems of many psychrotrophic microorganisms may cause further change in the nutritional composition of products after pasteurization, affecting the shelf-life quality of the products. Through the analysis of raw milk that had been stored for a long time, more types of genera that can grow at low temperatures were identified. The results differed from those of previous reports, and the enzyme systems such as proteases and lipases could also affect the quality of products after thermal processing.

A study on metabolite changes during the storage process of pasteurized milk based on metabolomics showed that the metabolite group remained stable during the first 8 days of refrigeration; metabolomic analysis after 8 days showed a decrease in the concentrations of pantothenic acid and butylcarnitine, whereas concentrations of some fatty acids, organic acids, α-AA, peptides, and ketones increased [23]. In this study, KEGG functional difference analysis based on functional annotation showed that, compared to the refrigerated raw milk, the refrigerated pasteurized milk had extremely significant positive statistical differences in metabolic pathways such as secondary metabolite biosynthesis, carbon metabolism, alanine, aspartic acid, and glutamic acid metabolism (amino acid metabolism), and glyoxylate and dicarboxylate metabolism (carbohydrate metabolism) (*p* < 0.001) (Figure 5). From metabolite-based KEGG functional difference analysis, it was found that the refrigerated pasteurized milk showed significant differences in glycerophoric metabolism (lipid metabolism), D-amino acid metabolism (other amino acid metabolism), lysine degradation (amino acid metabolism), and alpha-Linolenic acid metabolism (Figure 8). The results of functional analysis based on both the genetic and metabolite differences indicate that amino acid metabolism was more active during the pasteurization refrigeration process, showing that changes in the microbiota were the leading cause of enhanced amino acid metabolism. Compared with the analysis of the differential metabolites in the frozen raw milk, it was predicted that the metabolic pathways of the refrigerated pasteurized milk and the refrigerated raw milk were different. After pasteurization, significant metabolic shifts occurred alongside microbiota changes. Teichoic acid synthesis and glycerophospholipid metabolism were impacted in refrigerated raw milk (Figure 9). These pathways, linked to cell wall and membrane dynamics, indicate heightened cell membrane metabolism during refrigeration. Thermal stimulation could enhance this activity, possibly correlating with microbial stress resistance [33].

Previous research indicates that proteases from psychrotrophic bacteria can spoil UHT milk quality [34]. Recontamination during filling is another issue [35], as are variations in processing raw milk, which affect the microbial community in UHT milk [36]. Our study found that metagenomic data from three UHT milk samples were limited, with only one sample able to be successfully sequenced (Table 1). OTU analysis showed that microbial succession occurred in UHT milk, albeit minimally (Table S18). The varied distribution suggests that post-sterilization contamination affects UHT milk quality. Notably, *Rhodococcus*, *Phaffia*, and *Cellulomonas* are key genera in storage and warrant attention.

In summary, high-throughput metagenomic approaches were utilized to examine changes in the microbiomes of dairy products during their processing and storage periods. These analyses uncovered bacterial groups implicated in the quality-related changes in dairy products and identified a previously overlooked bacterial strain. Integration with the metabolomic analyses of the samples enabled the identification of the genera that were significantly influencing metabolite variations. The differential taxa originating from the initial microorganisms were likely the principal contributors to nutritional shifts during milk’s refrigeration. It is crucial to monitor the activity of the enzymes involved in amino acid metabolism, as they pertain to the growth and reproduction of microorganisms under refrigerated conditions. The lipid metabolic processes inferred from the metabolite difference analysis diverged from those deduced from genetic variance, suggesting that the lipid modifications in the sample may not correlate with shifts in the microbial community but could be linked to the intrinsic psychrophilic enzymes in the raw milk. Consequently, future research should also consider the activity of enzymes present in raw milk that are associated with lipid metabolism.

## 4. Materials and Methods

### 4.1. Samples Collection

Raw milk samples were obtained from Fengxian District, Shanghai Ranch, in December 2022. In accordance with the enterprise’s standard procedure for raw milk collection, the milk was stored at 4 °C and transported to undergo heat treatment at 75 °C for 15 s (pasteurization) and at 137 °C for 2–4 s (ultra-high temperature sterilization) using medium-scale equipment. The samples were collected in sterile 490-mL high-density polyethylene wide-mouthed bottles (Shitian, Shanghai, China) before and after processing. Both the raw and sterilized milk samples were delivered to the laboratory within 2 h and aseptically packaged into sterile 14 mL tubes (BD Falcon, MA, USA) for storage. The raw milk was aseptically divided into 12 samples; six were stored at −20 °C, while the other six were refrigerated at 4 °C. Concurrently, six pasteurized milk samples and six UHT milk samples were maintained at 4 °C. All samples were preserved for over a month prior to analysis. Subsequently, after centrifuging each sample at 12,000 rpm for 5 min, the precipitate and supernatant were separately placed in sterile centrifuge tubes. Precipitate samples were allocated for mNGS analysis, while supernatant samples were set for MS. All samples were preserved at −80 °C before analysis, with the details provided in Table 3. All the metagenomic sequencing experiments were conducted in triplicate and the metabolomic analysis experiments were performed on six replicates.

### 4.2. DNA Extraction, Library Construction, and mNGS

Four sets of precipitate samples, each containing three biological replicates, were subjected to mNGS. The Mag-Bind^®^ Soil DNA Kit (Omega Bio-tek, Norcross, GA, USA) was used to extract genomic DNA, following the manufacturer’s protocol. The concentration and purity of DNA were measured using TBS-380 (Turner Biosystems, Sunnyvale, CA, USA) and NanoDrop2000 (Thermo Fisher Scientific, Waltham, MA, USA), respectively. Furthermore, 1% agarose gel electrophoresis was used to evaluate the quality of the DNA extractions.

After fragmenting DNA to an average size of 400 bp using Covaris M220 (Covaris, Inc., Woburn, MA, USA), paired-end libraries were created with NEXTFLEX^®^ Rapid DNA Seq (Bio Scientific, Austin, TX, USA). Sequencing was performed on the Illumina NovaSeq platform (Illumina Inc., San Diego, CA, USA) provided by Majorbio Bio Pharm Technology Co., Ltd. (Shanghai, China).

### 4.3. Sequence Quality Control and Genome Assembly

Data were analyzed on Majorbio Cloud Platform (https://www.majorbio.com (accessed on 30 March 2023)). All reads were trimmed of adaptors and low-quality reads were removed using fastp [37]. They were then aligned to the *Bos taurus* genome using BWA [38], discarding any hits associated with the reads and their mated reads. The sequence that passed quality control was assembled using MEGAHIT v1.1.2 software, retaining contigs with minimum lengths of 300 bp.

### 4.4. Gene Prediction, Taxonomy, and Functional Annotation

The prediction of ORFs from every assembled contig was conducted using Prodigal v2.6.3 [39]/MetaGene [40] (http://metagene.cb.k.u-tokyo.ac.jp (accessed on 30 March 2023)). Subsequently, ORFs measuring ≥100 bp underwent translation into amino acid sequences through the application of the NCBI standard translation table (http://www.ncbi.nlm.nih.gov).

We predicted the gene sequences for all samples using the CD-HIT software v 4.7 [41]. Clustering was performed, with the parameters set to 90% identity and 90% coverage. From each cluster, we selected the longest gene sequence as representative and constructed a non-redundant gene set named GENESET_Origin. We obtained the base sequence of the genes within this non-redundant set. We employed the SOAPaligner software v2.21 [42] to align reads from each sample against the non-redundant gene set, with insertion fragment length parameters set to 500/300 base pairs, ensuring a 95% identity match. Subsequently, we calculated abundance-related data based on the number of reads for each gene in the corresponding samples. We aligned the representative sequences from GENESET_Origin to the following databases: NR (Non-Redundant Protein Sequence Database), as of September 2022; eggCOG (evolutionary genealogy of genes: Cluster of Orthologous Groups of proteins), as of 2020; and KEGG (Kyoto Encyclopedia of Genes and Genomes), as of September 2022. This alignment was performed using Diamond software v 0.8.35 [43], with an e-value cutoff of 1×10^−5^ used to obtain taxonomic, COG, and KEGG annotations. 

### 4.5. Analysis of Microbial Community Diversity

Based on the operational taxonomic units (OTUs) at the genus level, two alpha diversity indices, Chao1 (a richness estimator) and Shannon (a diversity index), were calculated, and a diversity index table for each sample was obtained.

The calculation formula used for the Chao index in this analysis was as follows:

(1) $$S_{chao1}=S_{obs}+\frac{n_{1}(n_{1}-1)}{2(n_{2}+1)}$$

*S_chao_*_1_ = the estimated number of OTUs;

*S_obs_* = the actual number of observed OTUs;

*n*_1_ = the number of OTUs containing only one sequence (such as “singletons”);

*n*_2_ = the number of OTUs containing only two sequences (such as “doubles”).

The calculation formula used for this analysis of the Shannon index was as follows:

(2) $$H_{shannon}=-\sum_{i=1}^{S_{obs}}\frac{n_{i}}{N}\ln\frac{n_{i}}{N}$$

where

*S_obs_* = the actual number of observed OTUs;

*n_i_* = the number of sequences contained in the i-th OTU;

*N* = the number of all sequences.

### 4.6. LC-MS Analysis

Four groups of supernatant samples, each containing six biological replicates, underwent LC-MS/MS nontargeted metabolomic analysis using Majorbio Bio-Pharm Technology Co., Ltd. (Shanghai, China). This was performed following standard procedures with some modification. Briefly, referencing the standard nontargeted metabolomic analysis method, we added an internal standard of L-2-chlorophenylalanine to the aforementioned sample and used extraction solution (acetonitrile-methanol = 1:1 (*v*:*v*)) for extraction [44]. LC-MS/MS analysis was conducted using the UHPLC-Q Exactive HF-X system (Thermo Scientific, Waltham, MA, USA) with an ACQUITY HSS T3 column (Waters, Milford, MA, USA) [45]. Analysis was performed in ESI+ or ESI- mode. QC samples were tested every 5–15 samples to ensure reliability and stability. We mixed extracts of all samples in equal volumes to prepare QC samples.

### 4.7. GC-MS Analysis

A precise volume of 100 μL of the liquid sample was measured for GC-MS spectrometry analysis. The preprocessing was carried out according to the reported literature [46].

GC-MS analysis used an Agilent 8890B-5977B (Agilent, Santa Clara, CA, USA) at Shanghai’s Majorbio Bio-Pharm. The instrument, equipped with a 70 eV EI source (Agilent, Santa Clara, CA, USA), employed a DB-5MS column and helium carrier gas at 1 mL/min. Starting at 60 °C for 30 s, the column temperature rose to 310 °C at 8 °C/min, a temperature held for 6 min. Samples were injected with 1 µL in split mode (15:1) at 260 °C. Mass spectrometry settings included a source temperature of 230 °C and quadrupole of 150 °C. Full scan mode covered m/z 50–500 at 3.2 scans/s.

Raw GC-MS data were processed with MassHunter v10.0 software, yielding a 3D CSV matrix of samples, metabolites, and intensities. The matrix was refined by removing internal standards and false positives, and by consolidating redundant peaks. Metabolites were identified using NIST (2017), Fiehn (2013), and MS-DIAL (2021) databases.

### 4.8. MS Data Analysis Processing and Annotation

LC-MS raw data preprocessing was conducted using Progenesis QI v3.0 software (Waters, MA, USA), resulting in a 3D CSV data matrix containing sample information, metabolite names, and mass spectral response intensities. Internal standard peaks and false-positive peaks were eliminated from the data matrix accordingly. Metabolites were identified by searching databases including the Human Metabolome Database (HMDB), Metlin (https://metlin.scripps.edu (accessed on 15 April 2023)), and the Majorbio Database.

Raw data obtained from GC-MS were preprocessed using the MassHunter Workstation Quantitative Analysis software (version v10.0.707.0), resulting in a 3D CSV data matrix containing sample information, metabolite name, and mass spectral response intensity. Internal standard peaks and false-positive peaks were removed from the data matrix accordingly. Metabolites were identified using primary public databases like NIST 2017, Fiehn 2013, and MS-DIAL 2021.

The data matrix, derived from the database search, was uploaded to the Majorbio cloud platform (https://cloud.majorbio.com (accessed on 10 May 2023)) for analysis. The initial preprocessing involved retaining at least 80% of metabolic features. For specific samples where metabolite levels were below the lower limit of quantification, the minimum metabolite value was estimated, and each metabolic signature was normalized relative to the total sum. To minimize errors arising from sample preparation and instrument variability, response intensities were normalized using the sum normalization method. Concurrently, variables of QC samples exhibiting a relative standard deviation (RSD) greater than 30% were discarded and log10-transformed, leading to the final data matrix for subsequent analyses. The data shown were the mean ± standard deviation (SD).

### 4.9. Statistical Analysis Methods

The data were analyzed using the free online platform of Majorbio cloud platform [47] (cloud.majorbio.com (accessed on 10 October 2023–10 January 2024)).

#### 4.9.1. Taxa and Metabolite Composition Analysis

We performed Venn plot analysis on the genera and metabolites that were common to multiple groups of samples and unique to a single group of samples, demonstrating the compositional similarity and overlap between the sample groups visually. Using the gene set GENESET-Origin data, we annotated, using the annotation table NR-Origin, and created a Venn plot of the structural composition of genera in different groups. We performed Venn plot analysis on the differential metabolites between the refrigerated pasteurized milk and the frozen raw milk, as well as the differential metabolites between the refrigerated and the frozen raw milk, to obtain the types and distribution of different metabolites in each group of samples.

#### 4.9.2. Taxa PCoA

PCoA was performed at the genus level based on the gene abundance tables, using the RPKM method for the abundance calculation. A two-dimensional visualization scatter plot was used to determine the similarities and differences in terms of communities between the control and treatment groups. The clustering degree of the sample community is reflected by the distance between samples, calculated using the Bray-Curtis algorithm.

#### 4.9.3. Difference Analysis

The differences between the Raw_fro and Raw_ref groups, as well as between the Raw_fro and Pas_ref groups, were analyzed based on taxa abundance information at the genus level, utilizing the Welch’s *t*-test (two-tailed test; significance level of 0.05). We used the *p* value calculation method to assess the false discovery rate (FDR); the confidence interval (0.95) was calculated using Welch’s inversion. Additionally, based on the KEGG annotation abundance information at the pathway ID level, the Welch’s *t*-test with the same parameters was employed for difference analysis between the Raw_ref and Pas_ref groups. The Kruskal-Wallis rank-sum test was conducted to compare multiple groups based on the sample grouping. The resulting *p* values were adjusted using the FDR method, and a post hoc Nemenyi test was employed for further statistical analysis. To analyze the significant differences among the selected sample groups, a graph was plotted showing the group names on the horizontal axis and the exponential mean on the vertical axis.

Principal component analysis (PCA) and OPLS-DA were performed using the R package “ropls” (Version 1.6.2), with a 7-cycle interactive validation. Metabolites with a VIP > 1 and *p* < 0.05 were identified as significantly different, based on OPLS-DA model VIP scores and *t*-test *p* values. The metabolites between groups were mapped into pathways through KEGG enrichment. The enrichment was then used to determine if a group of metabolites appeared in a functional node, extending single-metabolite annotation to a group.

The Python package “scipy.stats” was utilized to conduct pathway enrichment analysis on differential metabolic sets of Raw_ref_vs_Raw_fro and Pas_ref_vs_Raw_fro, employing Fisher’s exact test. The Benjamini and Hochberg (BH) multiple correction method was applied to validate the *p* value and manage false-positive outcomes. A *p* value threshold of 0.05 was set for characterizing the KEGG pathway as significantly enriched. A method using relative betweenness centrality topology was implemented to determine the relative significance of the pathways.

#### 4.9.4. Correlation Analysis

Based on the abundance of metabolites in each sample, the Euclidean distance algorithm was employed to compute the distance between two samples using the complete hierarchical clustering method, yielding a distance matrix, and the threshold of a *p* value was set as 0.05. Furthermore, the Pearson correlation coefficient was calculated to perform a visual sample correlation heatmap statistical analysis, which assesses the similarity within group samples and the differences between group samples. The positive and negative correlation thresholds were set as ≥0.8 and ≤−0.8, and the *p* value was set as <0.01. The correlation between differential metabolic sets and taxa abundance was also investigated using the aforementioned method, and the top 50 metabolite-genus correlations were displayed in the heatmap.

## Figures and Tables

**Table 1:** mNGS and assembly information.

Groups	Samples	Optimized Reads	Optimized Bases (bp)	Contigs	ORFs
Raw_fro	RAw_fro_1	53,867,630	8,116,741,291	70,515	142,253
	RAw_fro_2	55,008,670	8,286,484,529	64,916	135,320
	RAw_fro_3	63,800,856	9,608,917,943	67,471	143,276
Raw_ref	RAw_ref_1	62,946,434	9,490,863,131	70,997	154,713
	RAw_ref_2	71,983,366	10,849,612,040	72,332	160,636
	RAw_ref_3	63,740,996	9,604,086,372	71,333	153,063
Pas_ref	RP_ref_1	61,152,560	9,214,137,144	21,165	39,291
	RP_ref_2	58,224,872	8,774,437,192	23,589	41,496
	RP_ref_3	91,038,714	13,715,234,644	24,320	46,970
UHT_ref ^1^	RU_ref_2	4,349,358	654,056,765	14,633	53,055

^1^ Only one of the three parallel samples was successfully sequenced.

**Table 2:** The top 5 differentially accumulated metabolites of protein and fat metabolism with VIP values in 3 comparison groups.

Comparison Groups	Total Number of Differential Metabolites	Top Five Differential Metabolites	VIP ^1^	Relative Quantity of Raw_ref (RP_ref)	Relative Quantity of Raw_fro	Regulate	HMDB Subclass
Raw_ref vs. Raw_fro	462 (LC-MS)	Valylmethionine	2.4517	7.35 ± 0.06	1.41 ± 0.03	up	Amino acids, peptides, and analogues
		Biocytin	2.2861	7.12 ± 0.05	1.96 ± 0.03	up	Amino acids, peptides, and analogues
		PA(8:0/8:0)	2.1459	6.57 ± 0.09	2.03 ± 0.03	up	Glycerophosphates
		Pyridinoline	2.128	6.87 ± 0.17	2.21 ± 0.73	up	Amino acids, peptides, and analogues
		Hydroxytetradecenoylcarnitine	2.0905	6.24 ± 0.16	1.92 ± 0.03	up	Fatty acids and conjugates
	17 (GC-MS)	Citric Acid	2.889	4.17 ± 0.22	7.66 ± 0.04	down	Tricarboxylic acids and derivatives
		2,3-Butanediol	2.005	7.27 ± 0.14	5.58 ± 0.14	up	Alcohols and polyols
		Butane-2,3-diol	1.9093	7.29 ± 0.09	5.76 ± 0.11	up	Alcohols and polyols
		2-hydroxy-4-methylpentanoic acid	1.808	5.98 ± 0.10	4.61 ± 0.13	up	Fatty acids and conjugates
		L-Serine	1.7551	5.86 ± 0.86	4.28 ± 0.06	up	Amino acids, peptides, and analogues
RP_ref vs. Raw_fro	519 (LC-MS)	3b,6a-Dihydroxy-alpha-ionol 9-[apiosyl-(1->6)-glucoside]	2.6584	7.31 ± 0.39	0.64 ± 0.40	up	Fatty acyl glycosides
		PS(22:4(7Z,10Z,13Z,16Z)/22:5(7Z,10Z,13Z,16Z,19Z))	2.5301	5.93 ± 0.52	0.31 ± 0.71	up	Glycerophosphoserines
		11-Maleimidoundecanoic acid	2.5084	6.61 ± 0.34	1.16 ± 0.01	up	Fatty acids and conjugates
		Prolyl-Alanine	2.4519	6.09 ± 0.67	0.36 ± 0.02	up	Amino acids, peptides, and analogues
		3-[(2R)-2-Hydroxy-3-methyl-3-[(phosphonooxy)methyl]butanamido]propanoylcarnitine	2.449	6.37 ± 1.07	0.58 ± 0.02	up	Fatty acid esters
	22 (GC-MS)	D-Glucose	2.0015	7.34 ± 0.24	6.02 ± 0.08	up	Carbohydrates and carbohydrate conjugates
		Ethanamine	1.9081	5.41 ± 1.09	7.00 ± 0.06	down	Amines
		Butanoic acid	1.6228	5.38 ± 0.05	4.53 ± 0.07	up	Fatty acids and conjugates
		Gamma-Aminobutyric Acid	1.5961	6.21 ± 0.50	5.23 ± 0.13	up	Amino acids, peptides, and analogues
		2-hydroxyhexanoic acid	1.5905	5.32 ± 0.10	4.44 ± 0.29	up	Fatty acids and conjugates

^1^ All the VIP results in the table were statistically significant (*p* < 0.05).

**Table 3:** The highly correlated metabolites differentially accumulated metabolites in the refrigerated pasteurized milk.

Highly Correlated Metabolites	HMDB Subclass	Correlation			
		*Mriobacterium*	*Micrococcus*	*Acinetobacter*	*Pseudomonas*
Gln Leu Leu	–	0.8743	0.8456	−0.3182	−0.6086
Arg Leu	–	0.9686	0.9883	−0.8071	−0.939
13-Demethyl tacrolimus	Pyrimidines and pyrimidine derivatives	0.9683	0.9854	−0.8193	−0.9456
Levonorgestrel	Estrane steroids	0.9712	0.9938	−0.7845	−0.9236
S-(PGA1)-glutathione	Amino acids, peptides, and analogues	0.9782	0.9957	−0.7563	−0.9144
PC(PGF1alpha/20:2(11Z,14Z))	Not Available	0.9986	0.9609	−0.6973	−0.8746
Cucurbitacin I 2-glucoside	Steroidal glycosides	0.9751	0.9907	−0.6389	−0.8406
APC	Carbonyl compounds	0.8287	0.8517	−0.9142	−0.9413
2-Isopropylmalic Acid	Fatty acids and conjugates	0.8969	0.8692	−0.9454	−0.9729
Hydroxysepiapterin	Pterins and derivatives	0.892	0.9071	−0.9093	−0.9447
M-Coumaric acid	Phenylpropanoids and polyketides	0.9021	0.9043	−0.9253	−0.9873
PS(20:5(5Z,8Z,11Z,14Z,17Z)/16:0)	Glycerophosphoserines	0.8241	0.9162	−0.8303	−0.8884
Ppack	Amino acids, peptides, and analogues	0.9534	0.9183	−0.8924	−0.9792
5-(3-Pyridyl)-2-hydroxytetrahydrofuran	Not available	0.9316	0.9244	−0.9179	−0.9804
L-Lysine	Amino acids, peptides, and analogues	0.9223	0.9413	−0.901	−0.9792

## Data Availability

Data available from the corresponding authors upon reasonable request.

## Supplementary Materials

The following supporting information can be downloaded at: https://www.mdpi.com/article/10.3390/molecules29122745/s1, Figure S1: Sequence length distribution of non-redundant gene catalog; Figure S2: Taxa Fisher’s exact test of Raw_fro_1 sample and RU_ref_2 sample at the genus level; Figure S3: KEGG Pathway classification statistical bar chart; Figure S4: LC-MS QC sample evaluation chart; Figure S5: GC-MS QC sample evaluation chart; Figure S6: Correlation between genus abundance (Top 50) and differential metabolites (Top 50) in refrigerated pasteurized milk Table S1: Statistical table of the number and length of genes before and after redundancy removal; Table S2: Reads number; Table S3: Reads number_relative; Table S4: NR species annotation results; Table S5: Taxa abundance based on reads number; Table S6: Alpha diversity index results; Table S7: KEGG orthology abundance Table; Table S8: LC-MS metabolite analysis results (positive ionic mode); Table S9: LC-MS_MS metabolite analysis results (negative ionic mode); Table S10: GC-MS metabolite analysis results (positive ionic mode); Table S11: Differences of LC metabolites between groups RAw-ref group and RAw-fro; Table S12: Differences of LC metabolites between groups RP-ref group and RAW-fro group; Table S13: Differences of GC metabolites between groups RAw-ref group and RAw-fro; Table S14: Differences of GC metabolites between groups RP-ref group and RAW-fro group; Table S15: Unique differential metabolite set of refrigerated pasteurized milk; Table S16: Taxa abundance at genus (reads number-relative); Table S17: Correlation data Table of top 50 taxa and top 50 metabolites in refrigerated pasteurized milk; Table S18: differential metabolites analysis (UHT_ref_2_vs_Raw_fro_1).
