# Supplementary material for: Dynamic Interplay between Microbiota Shifts and Differential Metabolites during Dairy Processing and Storage
Source: Molecules. 2024 Jun 9;29(12):2745. doi: 10.3390/molecules29122745 (PMC11206652; doi:10.3390/molecules29122745)
Supplement: Supplementary file 1 [file molecules-29-02745-s001.zip › Supplementary Materials-Figures.pdf]

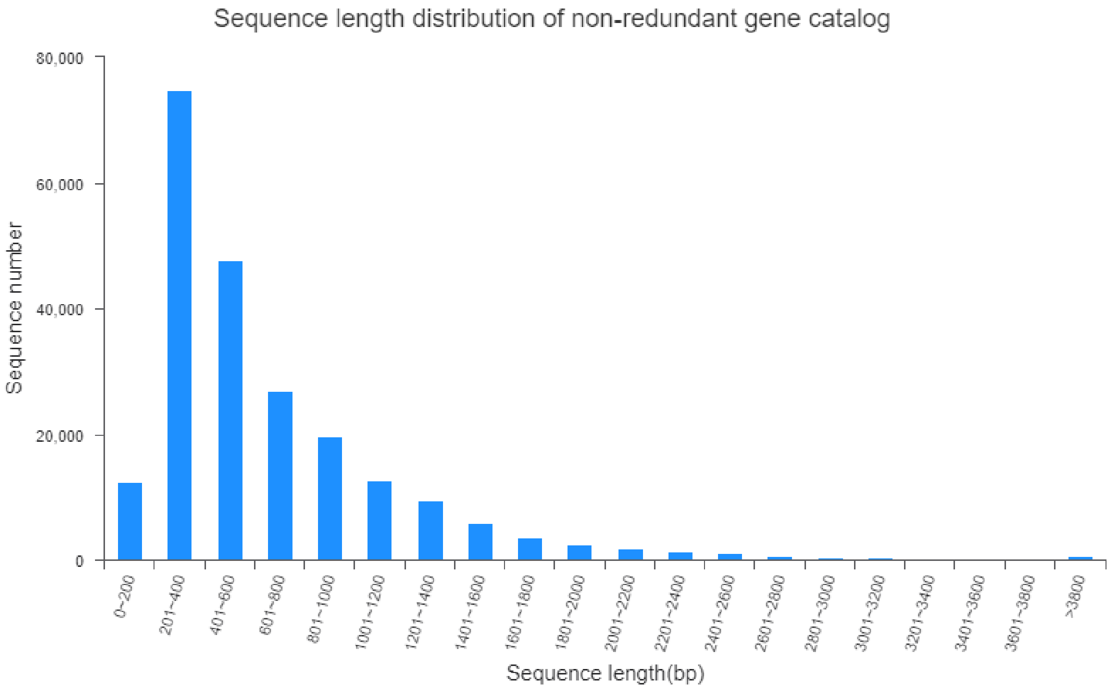

Figure S1. Sequence length distribution of non-redundant gene catalog

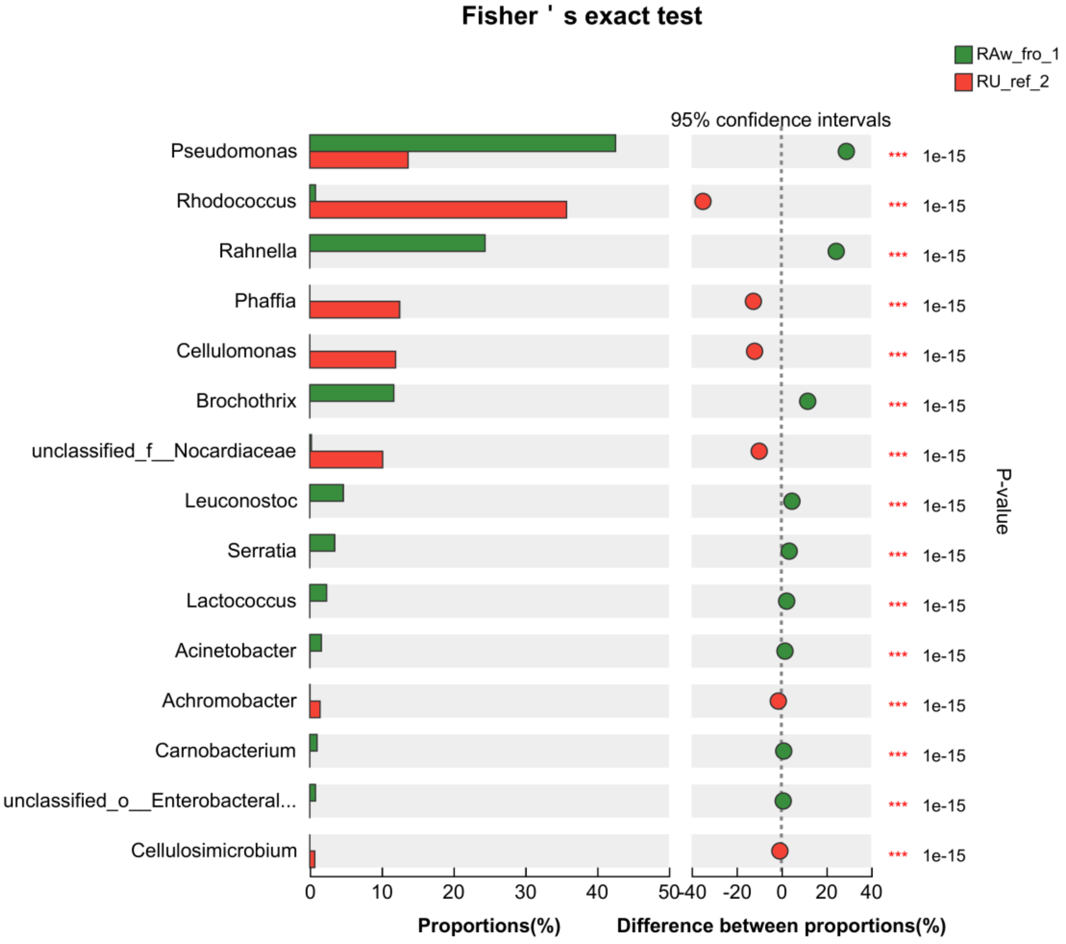

Figure S2. Taxa Fisher′s exact test of Raw_fro_1 sample and RU_ref_2 sample at the genus level

The difference between Raw_fro_1 sample and RU_ref_2 sample, were analyzed. Based on taxa abundance information at the genus level, the Fisher's exact test (two tailed test; significance level=0.05; P-value calculation method: FDR) were performed for the difference analysis between two samples.

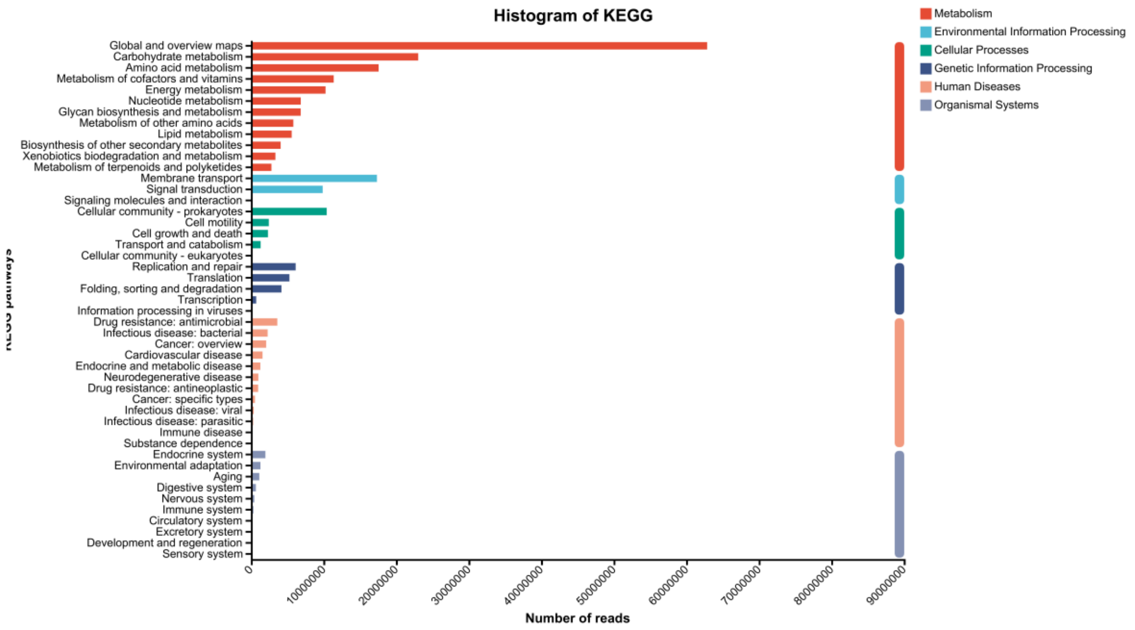

Figure S3. KEGG Pathway classification statistical bar chart

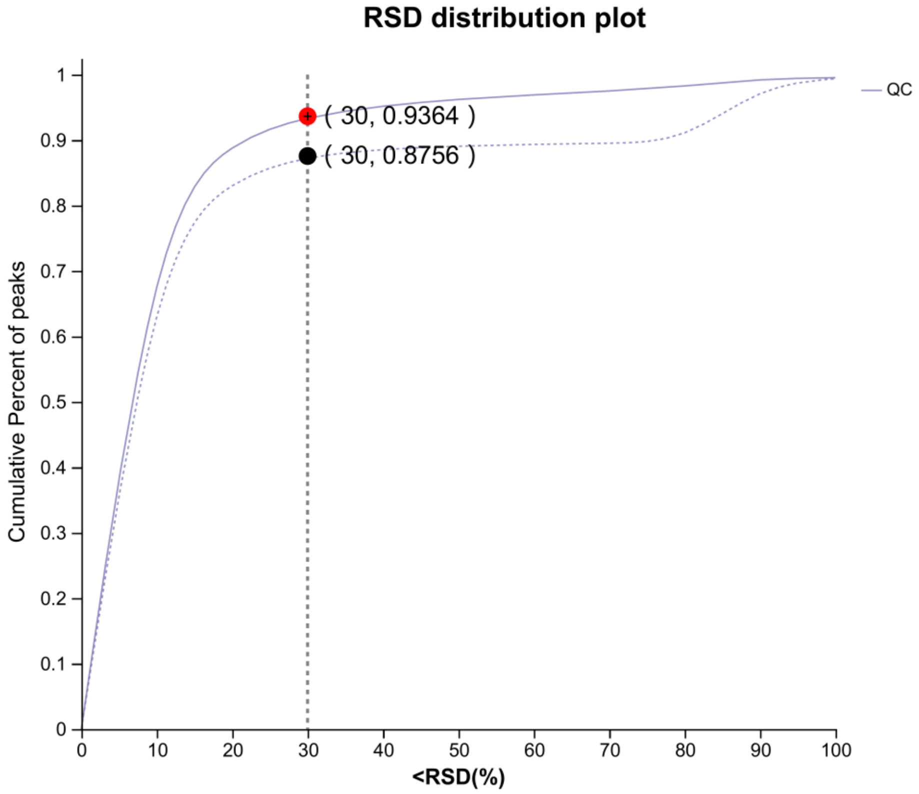

Figure S4. LC-MS QC sample evaluation chart

The dashed line represents before preprocessing, and the solid line represents after preprocessing. For the overall data, when RSD<0.3, the cumulative proportion of peaks>70%, the overall data is qualified.

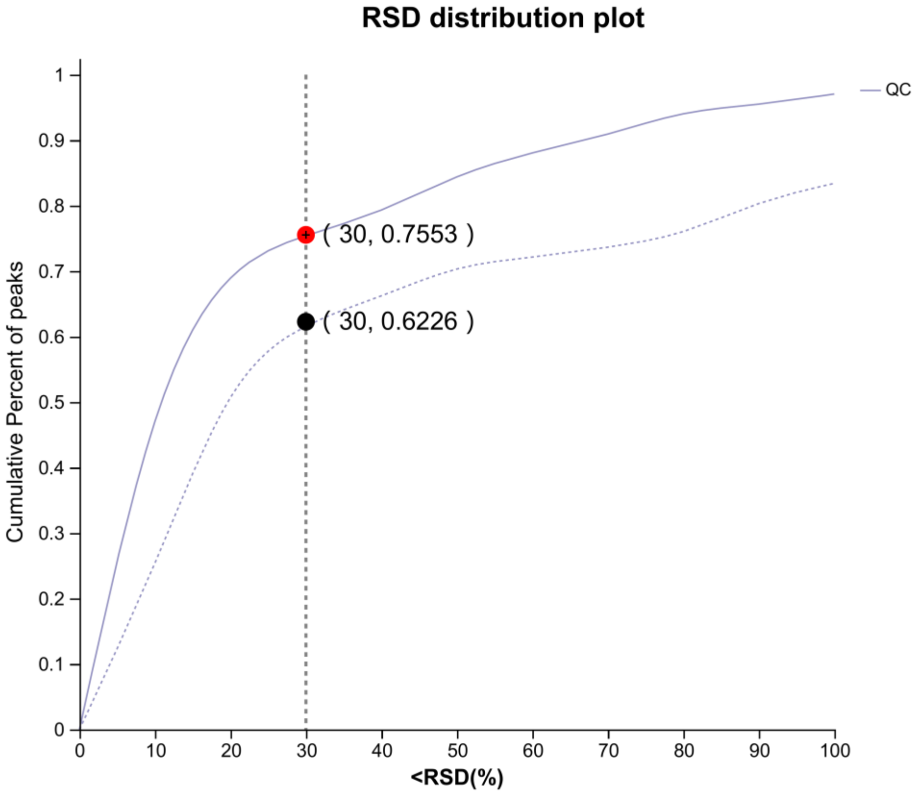

Figure S5. GC-MS QC sample evaluation chart

The dashed line represents before preprocessing, and the solid line represents after preprocessing. For the overall data, when RSD<0.3, the cumulative proportion of peaks>70%, the overall data is qualified.

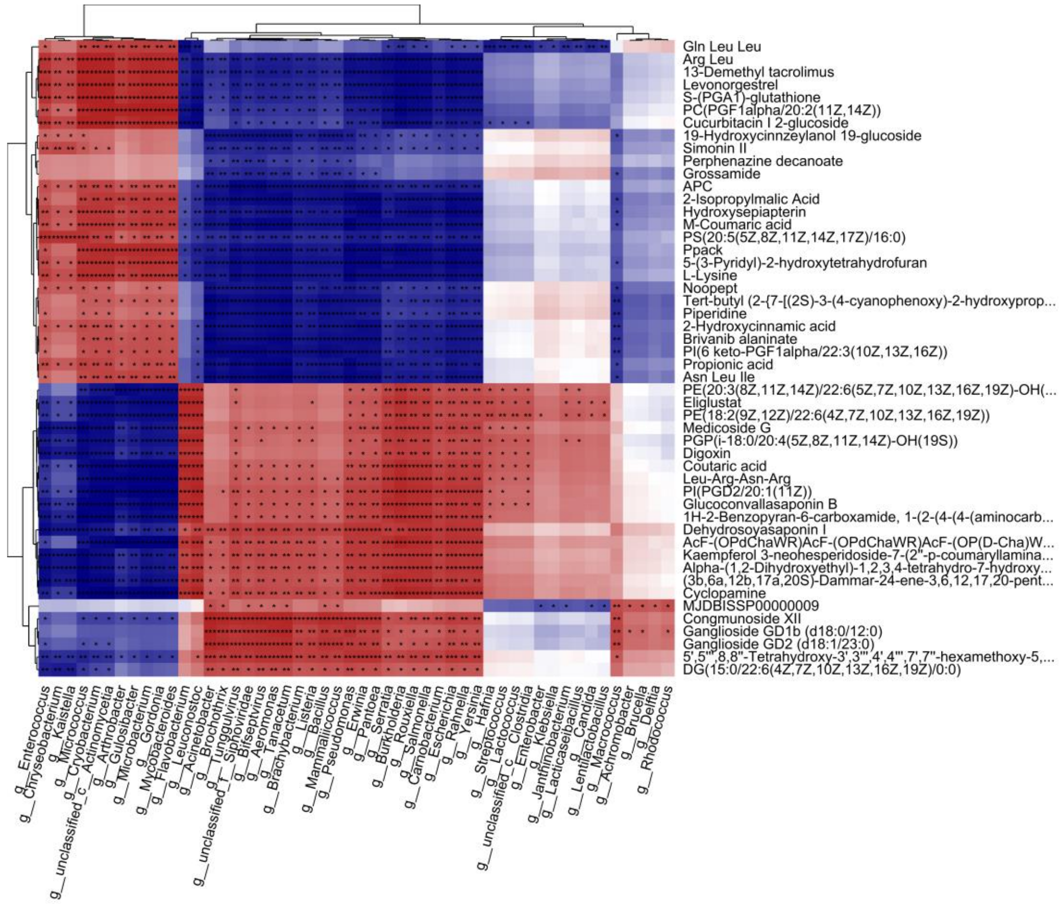

Figure S6. Correlation between genus abundance (Top 50) and differential metabolites (Top 50) in refrigerated pasteurized milk
