# Supplementary figures and images for: Dynamic Interplay between Microbiota Shifts and Differential Metabolites during Dairy Processing and Storage
Source: Molecules. 2024 Jun 9;29(12):2745. doi: 10.3390/molecules29122745 (PMC11206652; doi:10.3390/molecules29122745)

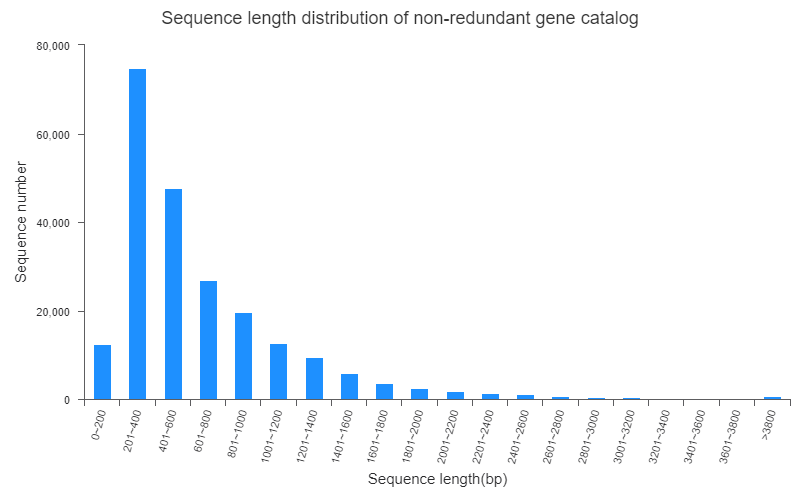

Supplement: Supplementary file 1 [file molecules-29-02745-s001.zip › Figure S1 Sequence length distribution of non-redundant gene catalog(GENESET_Origin).png]

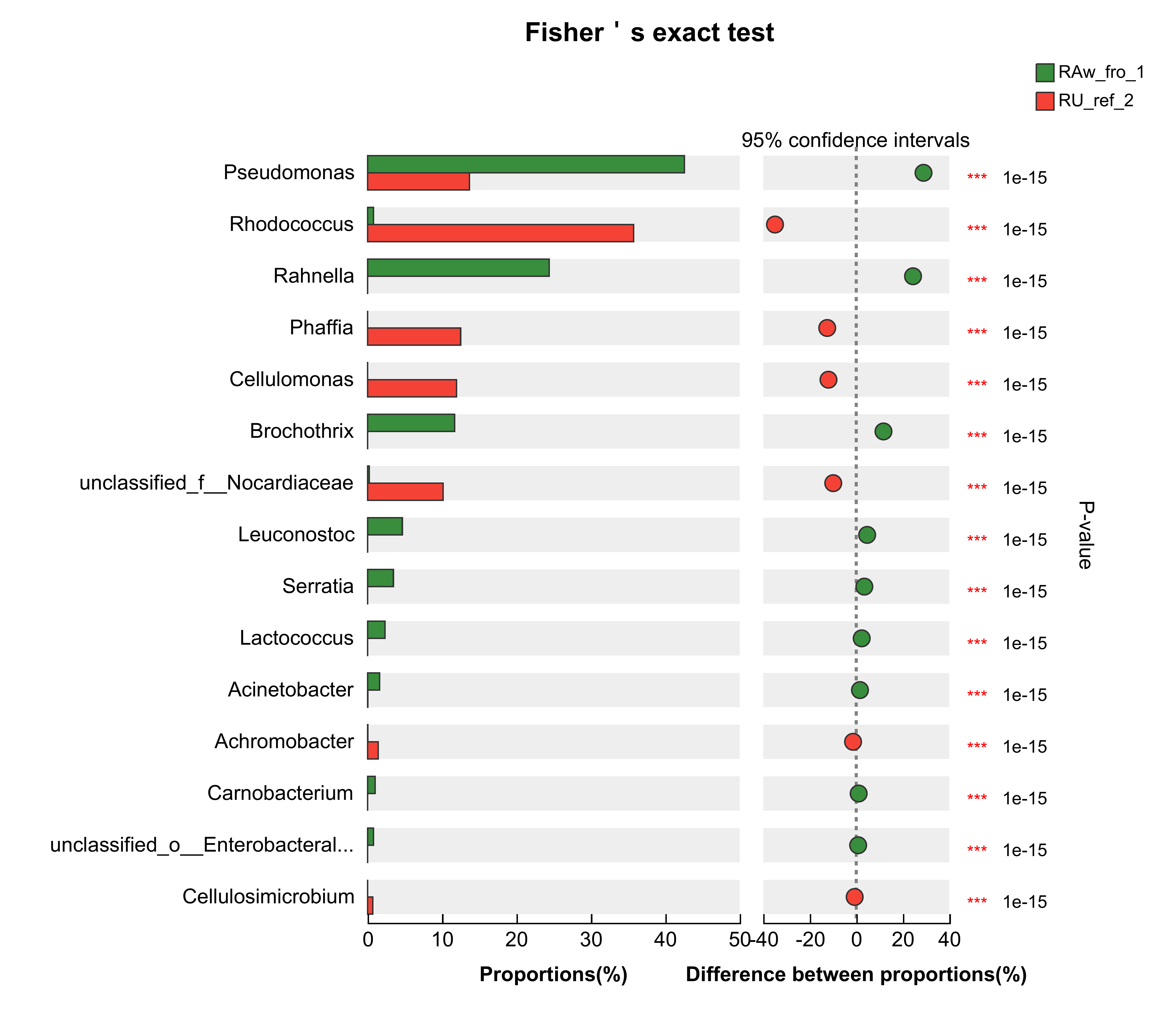

Supplement: Supplementary file 1 [file molecules-29-02745-s001.zip › Figure S2 Taxa Fisher's exact test of Raw_fro_1 sample and RU_ref_2 sample at the genus level.png]

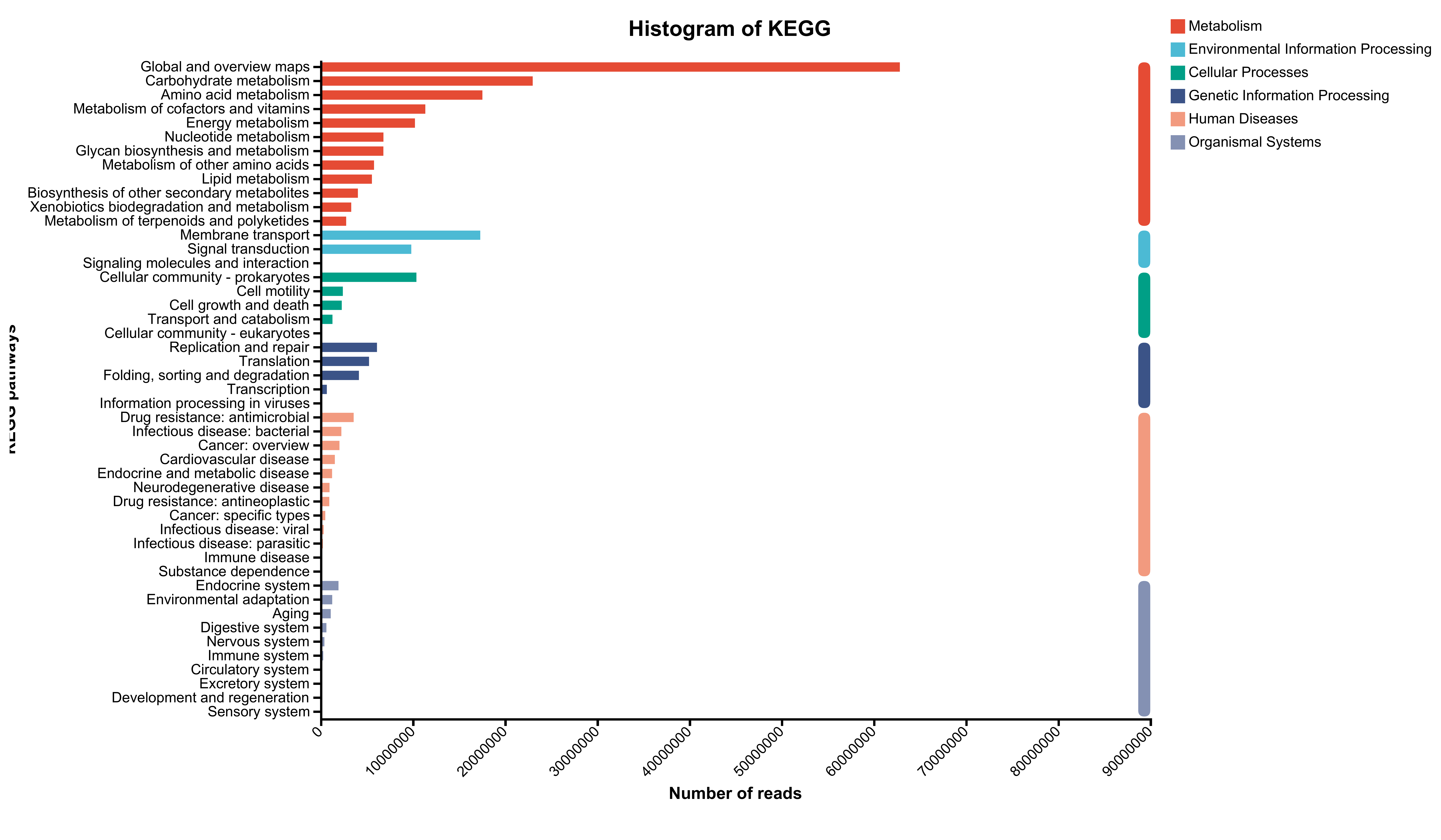

Supplement: Supplementary file 1 [file molecules-29-02745-s001.zip › Figure S3 KEGG Pathway classification statistical bar chart.png]

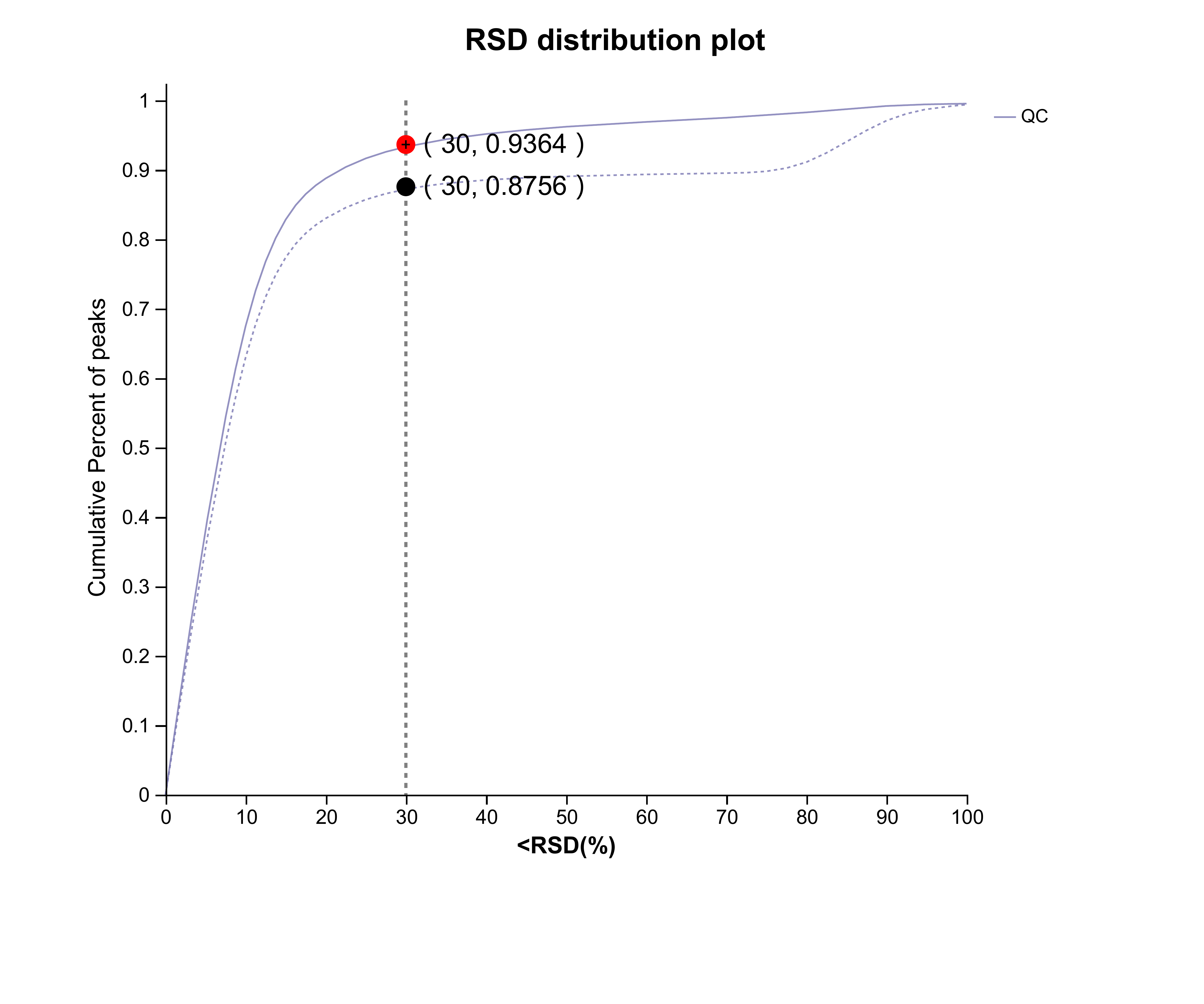

Supplement: Supplementary file 1 [file molecules-29-02745-s001.zip › Figure S4 LC-MS QC sample evaluation chart.png]

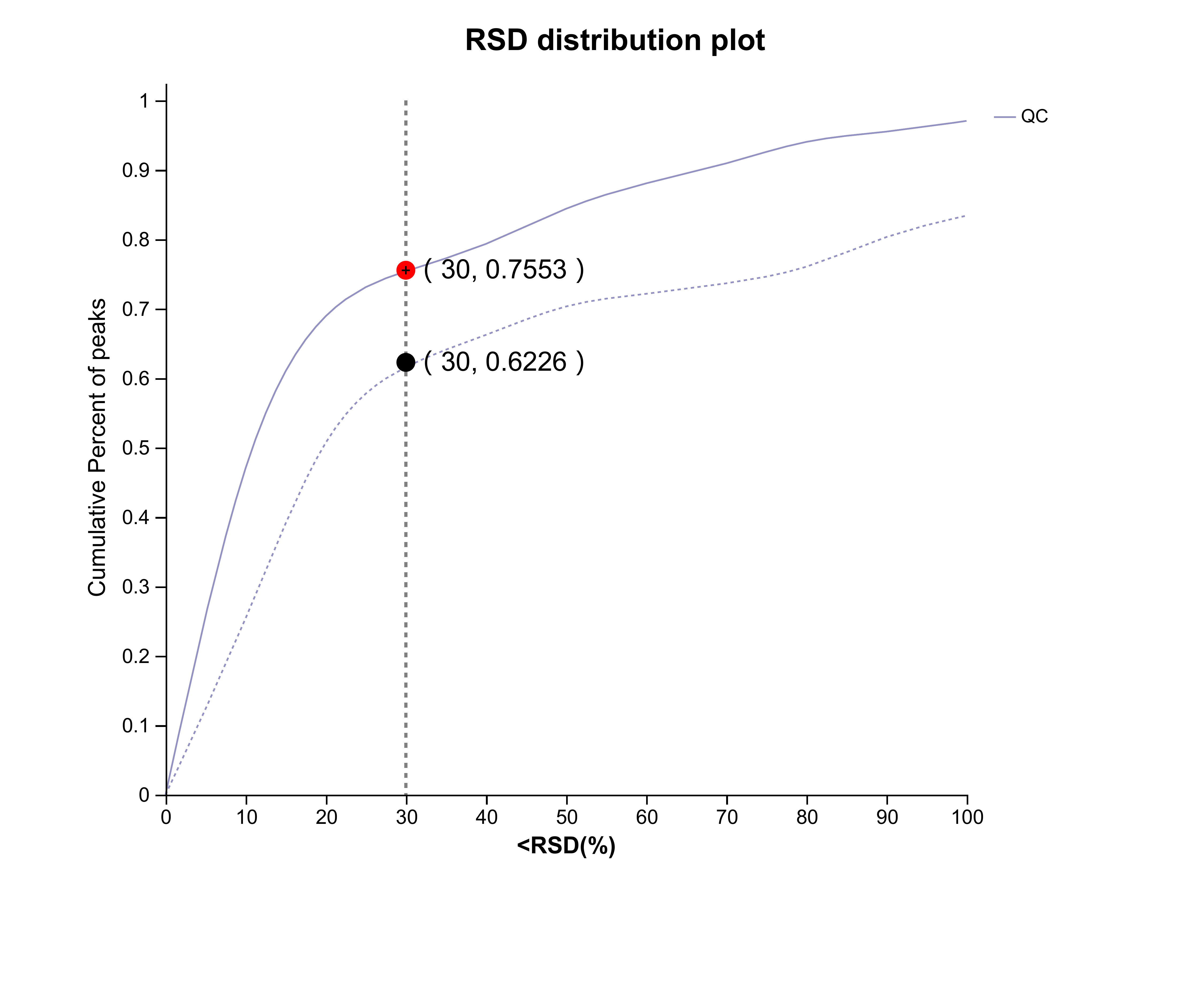

Supplement: Supplementary file 1 [file molecules-29-02745-s001.zip › Figure S5 GC-MS QC sample evaluation chart.png]

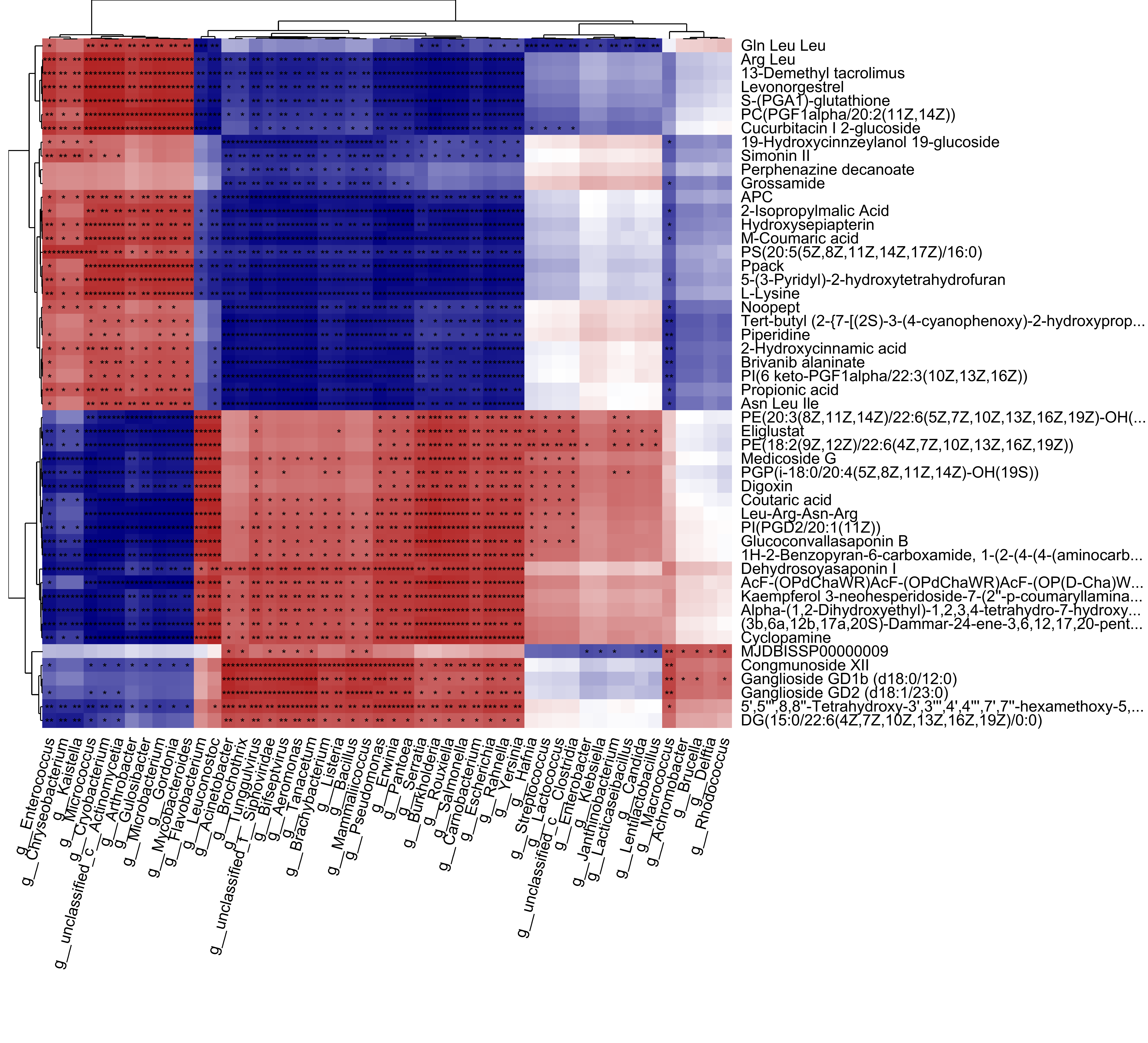

Supplement: Supplementary file 1 [file molecules-29-02745-s001.zip › Figure S6 Correlation between genus abundance (Top 50) and differential metabolites (Top 50) in refrigerated pasteurized milk.png]
